# Metabolomics and network pharmacology reveal partial insights into the hypolipidemic mechanisms of ferulic acid in a dyslipidemia mouse model

**DOI:** 10.3389/fphar.2024.1466114

**Published:** 2024-09-20

**Authors:** Zhihao Zeng, Guanlin Xiao, Yanchang Liu, Minshan Wu, Xingqin Wei, Canhui Xie, Guangying Wu, Dezheng Jia, Yangxue Li, Sumei Li, Xiaoli Bi

**Affiliations:** ^1^ School of the Fifth Clinical Medicine, Guangzhou University of Chinese Medicine, Guangzhou, China; ^2^ Guangdong Provincial Engineering Technology Research Institute of Traditional Chinese Medicine/Guangdong Provincial Key Laboratory of Research and Development in Traditional Chinese Medicine, Guangzhou, China

**Keywords:** ferulic acid, hyperlipidemia, metabolomics, network pharmacology, conjoint analysis

## Abstract

**Introduction:**

Hyperlipidemia is a condition characterized by abnormal levels of lipids and lipoproteins in the plasma, posing significant health risks. Ferulic acid (FA) is an organic acid with therapeutic properties for diabetes and hyperlipidemia.

**Methods:**

To explore biomarkers for FA treatment of hyperlipidemia and elucidate the mechanisms of lipid-lowering-related changes in metabolic pathways by metabolomics and network pharmacology. Initially, a hyperlipidemic mouse model induced by triton WR-1339 was established to evaluate the therapeutic effects of FA. Subsequently, serum metabolomics was utilized to identify differential metabolites, and metabolic pathway analysis was performed using MetaboAnalyst 6.0. Thirdly, network pharmacology was employed to identify potential targets of FA for hyperlipidemia. Finally, the compound-target-metabolite (C-T-M) network obtained core targets and validated them with molecular docking.

**Results:**

Biochemical analysis and histological examination showed that FA had lipid-lowering effects on hyperlipidemic mice. It identified 31 potential biomarkers for FA against hyperlipidemia by metabolomics involving lipid and amino acid metabolism. Lipid and atherosclerosis signaling pathways were identified as the key signaling pathways of FA against hyperlipidemia by KEGG analysis. Conjoint analysis showed that FA against hyperlipidemia was associated with 18 core targets and six biomarkers. Molecular docking results showed that FA has a high binding affinity to these core targets.

**Discussion:**

Through the synergy of network pharmacology and metabolomics, this study provides insights into how FA regulates endogenous metabolites, underscoring its promise as a treatment for hyperlipidemia.

## 1 Introduction

Hyperlipidemia is a disorder resulting from the buildup of plasma lipids and lipoproteins and is characterized by elevated triglycerides (TG), total cholesterol (TC), and low-density lipoprotein-cholesterol (LDL-c) but decreased high-density lipoprotein-cholesterol (HDL-c) (Das and Kumar, 2021). One of the aetiological risk factors for the development of cardiovascular diseases (CVDs) is hyperlipidemia. CVDs are the main cause of death in adults, and those with hyperlipidemia have a twofold increased risk of CVDs (Karr, 2017), which places a heavy economic burden on society. The risk of CVDs escalates with TG concentrations ranging from 2 to 10 mmol/L, and concentrations exceeding 10 mmol/L also increase the likelihood of acute pancreatitis and potentially CVDs (Nordestgaard and Varbo, 2014). Currently, the majority of hyperlipidemia-related illnesses are treated clinically with statins, ezetimibe, and fibrates. Nevertheless, there are certain side effects, including statin-induced myopathy and loss of liver function (Abd and Jacobson, 2011; Aguilar-Salinas et al., 2022; Yamashita et al., 2020). Therefore, there is considerable potential for developing safe and efficacious alternative medications for treating hyperlipidemia.

Ferulic acid (FA), a phenolic compound widely found in plants, is recognized for its antioxidant and anti-inflammatory properties. It has been reported to inhibit oxidative stress and apoptosis, and has shown a potential to reduce the progression of diabetes and hyperlipidemia (Bumrungpert et al., 2018; Mei et al., 2023; Neto-Neves et al., 2021; Zhu et al., 2022). Studies have demonstrated that FA can lower lipid levels in rats with hyperlipidemia induced by a high-fat diet or triton (Jain and Surana, 2016; Nouni et al., 2023). However, FA regulates lipid metabolism in hyperlipidemia, but its mechanism for maintaining body homeostasis remains unclear.

In this study, we integrated network pharmacology and metabolomics strategies to investigate the mechanism of FA in hyperlipidemic mice. Firstly, metabolomics was analyzed by UPLC-Q-TOF-MS/MS, and data was processed through the MetaboAnalyst website and ultimately screened relevant metabolic pathways and potential biomarkers. In addition, core targets and pathways of FA for hyperlipidemia were screened by network pharmacology. Ultimately, we analyzed the targets and biomarkers in conjunction with metabolomics and network pharmacology and then analyzed the binding of FA to the core targets using molecular docking to elucidate the mechanism of FA in the treatment of hyperlipidemia.

## 2 Materials and methods

### 2.1 Materials and animals

Ferulic acid (CAS: 1135-24-6, batch number: 110773-201614) was obtained from the National Institute for Food and Drug Control (Beijing, China). Triton WR-1339 was obtained from Sigma-Aldrich (Shanghai, China). Twenty-four male Kunming (KM) mice (18–22 g body weight) were purchased from the Guangdong Medical Laboratory Animal Center (Guangzhou, China) (Permit number: 44007200106576). All animal experiment procedures were performed strictly with the Laboratory Animal Guideline for Ethical Review of Animal Welfare (GB/T 35892-2018, China) and were approved by the Guangdong Provincial Engineering Technology Institute of Traditional Chinese Medicine (Guangzhou, China).

### 2.2 Animal experiments

**FIGURE 1:**
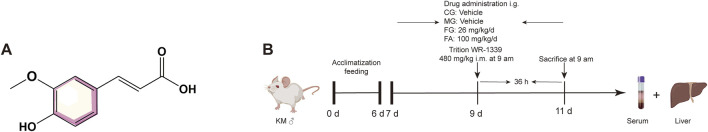
Chemical structure of FA **(A)**. Scheme of FA administration and triton WR-1339 injection **(B)**.

The standard conditions of temperature, humidity, and light were used to acclimatize all animals for a week. The KM mice were randomly divided into the following 4 groups of 6 mice in each group: control group (CG), model group (MG), fenofibrate group (FG, 26 mg/kg) (Li et al., 2020), and ferulic acid group (FA, 100 mg/kg) (Kesh et al., 2013). The administration groups were given corresponding drugs by gavage and once a day for 5 days. On the third day of administration, all groups were intramuscularly administered triton WR-1339 (480 mg/kg) to build an acute hyperlipidemia model excluding the CG. On the fifth day, after administrating for 1 h, all mice were anesthetized with isoflurane and sacrificed through inner canthus artery exsanguination. Blood samples were preserved at room temperature and centrifuged at 3,000 rpm at 4°C for 15 min to isolate the serum. Serum and liver were saved at −80°C until the next steps. The flow chart is shown in Figure 1.

### 2.3 Biochemistry analysis

The serum’s TC, TG, HDL-c, and LDL-c contents were detected using commercial reagent kits (Nanjing Jiancheng Bioengineering Institute, Nanjing, China) according to the manufacturer’s instructions.

### 2.4 Histological examinations

Mice liver tissues were immobilized with 4% paraformaldehyde solution, then dehydrated, embedded in paraffin, and sliced into 4 μm thick sections for H&E staining, and the histomorphology of the livers was observed and recorded using a microscope.

### 2.5 Serum and quality control samples preparation

Serum (100 μL) was mixed with 400 μL of pre-cooled acetonitrile-water mixture (1∶1, v/v) and vortexed for 30 s. The samples were incubated at −20°C for 1 h to precipitate the protein, then centrifuged for 15 min (4°C, 12,000 rpm), transfered the supernatant (400 μL) to a new 1.5 mL tube and blown dry with nitrogen. 100 μL of pre-cooled acetonitrile-methanol mixture (1∶1, v/v) was added into samples and vortexed for 30 s, centrifuged for 15 min (4°C, 12,000 rpm), and accurately absorbed 80 μL of the supernatant into the injection bottle for the next analysis.

Quality control (QC) samples were prepared by mixing 10 μL aliquots of each analyzed sample. To test the stability of UPLC-Q-TOF-MS/MS, QC samples were injected once every six samples during the metabolomics (Xiao et al., 2023).

### 2.6 Serum metabolomics analysis

The serum metabolomics analysis was carried out by Agilent 1,290 II coupled to AB SCIEX X500R Q-TOF-MS/MS. The chromatographic separations were achieved on a Waters UPLC BEH C18 column (2.1 mm × 100 mm, 1.7 μm), flow rate 0.3 mL/min, injector temperature 4°C, column temperature 35°C, injection volume 1 μL. Mobile phase: gradient elution of acetonitrile (A) - 0.1% formic acid water (B) (0–1 min, 2% A; 1–3 min, 2%–10% A; 3–7 min, 10%–40% A; 7–16 min, 40%–75% A; 16–20 min, 75%–98% A; 20–23 min, 98% A). MS was performed in positive and negative ion modes with electrospray ionization (ESI). The optimization source parameters were set as follows: ion voltage −4,500 V and +5,500 V, Gas1 55 psi, Gas2 55 psi, curtain gas 35 psi, de-clustering potential voltage 60 V, ion source temperature 500°C, collision energy 35 V, collision energy spread 15 V, full scan *m*/*z* 50-1,000.

### 2.7 Data processing and potential biomarkers selection

Multivariate statistical analysis was conducted using SIMCA 14.1 software with unsupervised principal component analysis (PCA) and orthogonal partial least discriminant analysis (OPLS-DA) models, and the results of OPLS-DA needed to be further confirmed by 200 times permutation evaluations. Statistical analyses were then performed using the online tool Metaboanalyst 6.0 for univariate student *t*-test and finally for difference multiple (FC value) analysis. The metabolites with significantly different (Variable important in projection (VIP) > 1, *p* < 0.05, Fold change (FC) < 0.8, or FC > 1.2) between MG and CG and between FA and MG were screened for preliminary identification and analysis. Further analysis of these metabolites was performed to screen for significant differences between FA and MG metabolites. It showed a recovery trend compared with CG, which was screened as the differential metabolite for treating hyperlipidemia. Potential biomarkers were characterized according to the METLIN and Human Metabolome databases, and pathways of differential metabolites were analyzed using the online software MetaboAnalyst 6.0 (http://www.metaboanalyst.ca/) (Pang et al., 2024).

### 2.8 Network pharmacology

#### 2.8.1 Collection of FA and hyperlipidemia-related targets

The structure and SMILE nodes of FA were determined by searching for “ferulic acid” and “fenofibrate” on the PubChem database (https://pubchem.ncbi.nlm.nih.gov/), respectively. Based on the results, potential targets of FA and fenofibrate were retrieved from the ChEMBL database (Nowotka et al., 2017) with the limited species of “*Homo sapiens*”. Then, we uploaded the FA and fenofibrate targets obtained to the Uniport database (UniProt Consortium, 2023) for standardization. We also searched the relevant targets through the GeneCard (https://www.genecards.org/), Comparative Toxicogenomics Database (CTD, https://ctdbase.org/), and Drugbank (https://www.drugbank.com/) databases with the keyword “hyperlipidemia” and limited the species to “*Homo sapiens*”. The related potential targets were screened by merging and deduplicating.

#### 2.8.2 Protein-protein interaction (PPI) network construction and analysis

Venny 2.1.0 (https://bioinfogp.cnb.csic.es/tools/venny/) screened the overlapping targets between FA and hyperlipidemia. Then, the Search Tool for the Retrieval of Interacting Genes/Proteins (STRING, https://string-db.org/) was used to predict the interaction between proteins. According to the previous results, the overlapping targets were analyzed on STRING to create a protein-protein interaction network (PPI network), Cytoscape 3.7.2 was then used to visualize a PPI network.

#### 2.8.3 Gene ontology (GO) enrichment and Kyoto Encyclopedia of Genes and Genomes (KEGG) pathway enrichment

To further explore the biological function of potential targets of FA and hyperlipidemia, the Bioconductor clusterProfiler and org. Hs.eg.db package of R 4.0.2 (https://cran.r-project.org/src/base/R-4/) software and DOSE packages (Yu et al., 2015; Zou et al., 2019) performed Kyoto Encyclopedia of Genes and Genomes (KEGG) pathway enrichment analysis (Lin et al., 2021) and Gene Ontology biofunctional enrichment analysis (Gene Ontology Consortium, 2015), including assessment of biological processes (BP), cellular components (CC) and molecular functions (MF) to elucidate the significant biological functions of FA and hyperlipidemia. After inputting the data of potential targets into R 4.0.2, we set the threshold as *p* < 0.05 and *q* < 0.05 to ensure the main pathway of the obtained targets.

Furthermore, we performed KEGG enrichment analysis on the overlapping targets of FA and hyperlipidemia using R 4.0.2 software. This approach elucidates critical signaling pathways central to the biological processes influenced by FA in the context of hyperlipidemia.

### 2.9 Integrated analysis of metabolomics and network pharmacology

To further explore the hypolipidemic mechanism of FA on hyperlipidemia from upstream to downstream signals, MetaboAnalyst 6.0 was used to perform an integrated analysis of metabolomics and network pharmacology. By constructing a compound-target-metabolite (C-T-M) network, we identified core targets shared between metabolite-associated targets and potential therapeutic targets. These core targets represent key entities through which FA exerts its therapeutic effects in treating hyperlipidemia. The conjoint analysis for fenofibrate was the same as above.

### 2.10 Molecular docking

We utilized molecular docking to elucidate the interactions between FA and core target proteins by predicting their binding modes and affinities. The crystal structure format files for both FA and core targets were downloaded from the PubChem database and the RCSB Protein Data Bank (PDB, https://www.rcsb.org/), respectively. Removed water molecules from core proteins with AutoDockTools 1.5.6 software, added nonpolar hydrogens, and calculated the Gasteiger charges. The ligands and receptors were docked in PDBQT format via AutoDock Vina (Trott and Olson, 2010).

### 2.11 Statistical analysis

Herein, the results were shown as the mean ± SD. Statistical analysis was carried out by one-way ANOVA, student’s *t*-test was applied to compare two groups, and significance was established by GraphPad Prim 9.0. Metabolomics data are normalized by MetaboAnalyst 6.0 and imported into SIMCA 14.1 for further steps. We used the following annotations to indicate statistical significance: ^#^ *p* < 0.05, ^##^ *p* < 0.01, *p < 0.05, and **p < 0.01.

## 3 Results

### 3.1 Hypolipidemic activity of FA

**FIGURE 2:**
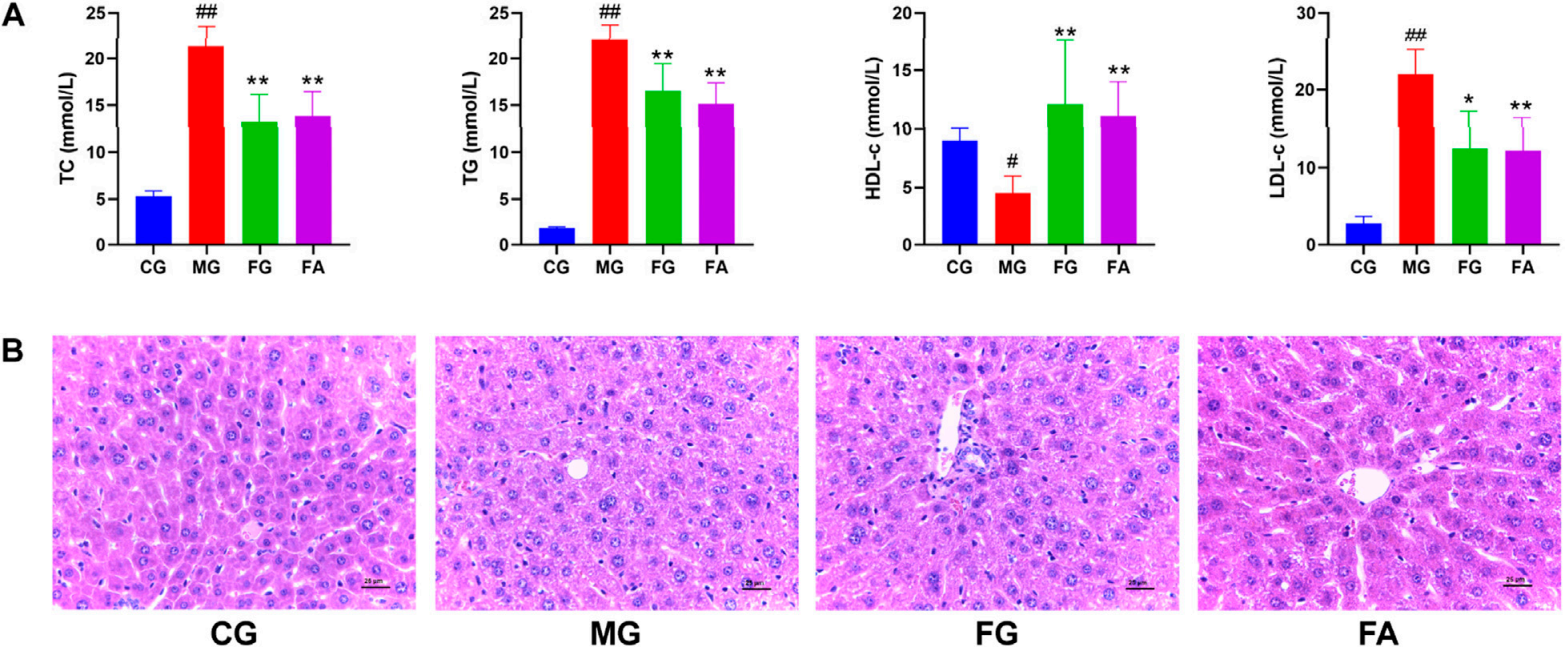
Effect of FA on serum and liver in triton WR-1339-induced hyperlipidemia mice. Biochemical analyses of serum TC, TG, HDL-c, and LDL-c **(A)**. H&E staining of the liver (×400) **(B)**. ^#^ *p* < 0.05, ^##^ *p* < 0.01 vs CG; ^*^ *p* < 0.05, ^**^ *p* < 0.01 vs MG.

We determined serum levels of total cholesterol (TC), triglycerides (TG), high-density lipoprotein cholesterol (HDL-c), and low-density lipoprotein cholesterol (LDL-c) as depicted in Figure 2A. The serum TC, TG, and LDL-c levels were significantly elevated in the MG compared to the CG. However, both the FG and FA groups exhibited significant reductions in TG, TG, and LDL-c levels post-treatment. Conversely, HDL-c levels were significantly lower in the MG than in the CG, but markedly higher in both the FG and FA groups. These findings indicate that FA effectively mitigated dyslipidemia in hyperlipidemic mice.

As observed by H&E staining results, the hepatocytes of CG were well arranged with intact nuclei and no inflammatory cell infiltration or vacuolar lesions, while MG had disorganized hepatocytes with unclear cell borders, some missing nuclei, diffuse steatosis of hepatocytes, and increased vacuolar lesions and inflammatory infiltration. Compared with MG, FG and FA had well-arranged hepatocytes, significantly fewer fat vacuoles, and improved cell morphology, although a small amount of inflammatory infiltration was still present (Figure 2B).

### 3.2 Metabolomics profiling

**FIGURE 3:**
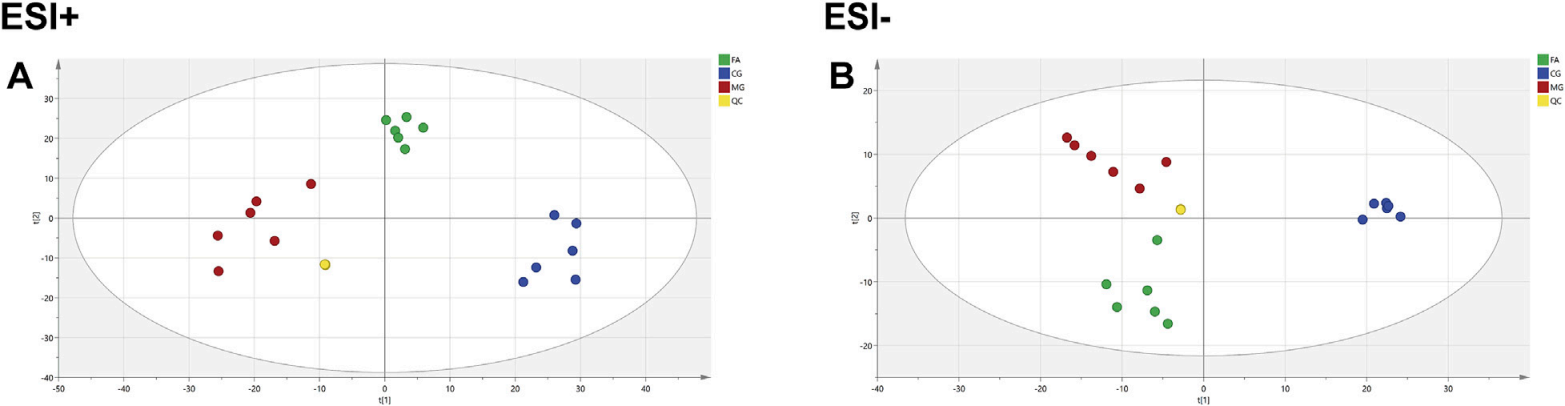
PCA diagram for CG, MG, and FA in the positive ESI mode **(A)** and negative ESI mode **(B)**.

QC samples are mixtures of each sample and are primarily used to assess the accuracy, stability, and reproducibility of experiments and to ensure the reliability of results. As shown in Figures 3A, B, the QC samples displayed good clustering in the PCA score plot, demonstrating that the preparation and experimental conditions of the samples were consistent and reliable, affirming the accuracy of the acquired data.

**FIGURE 4:**
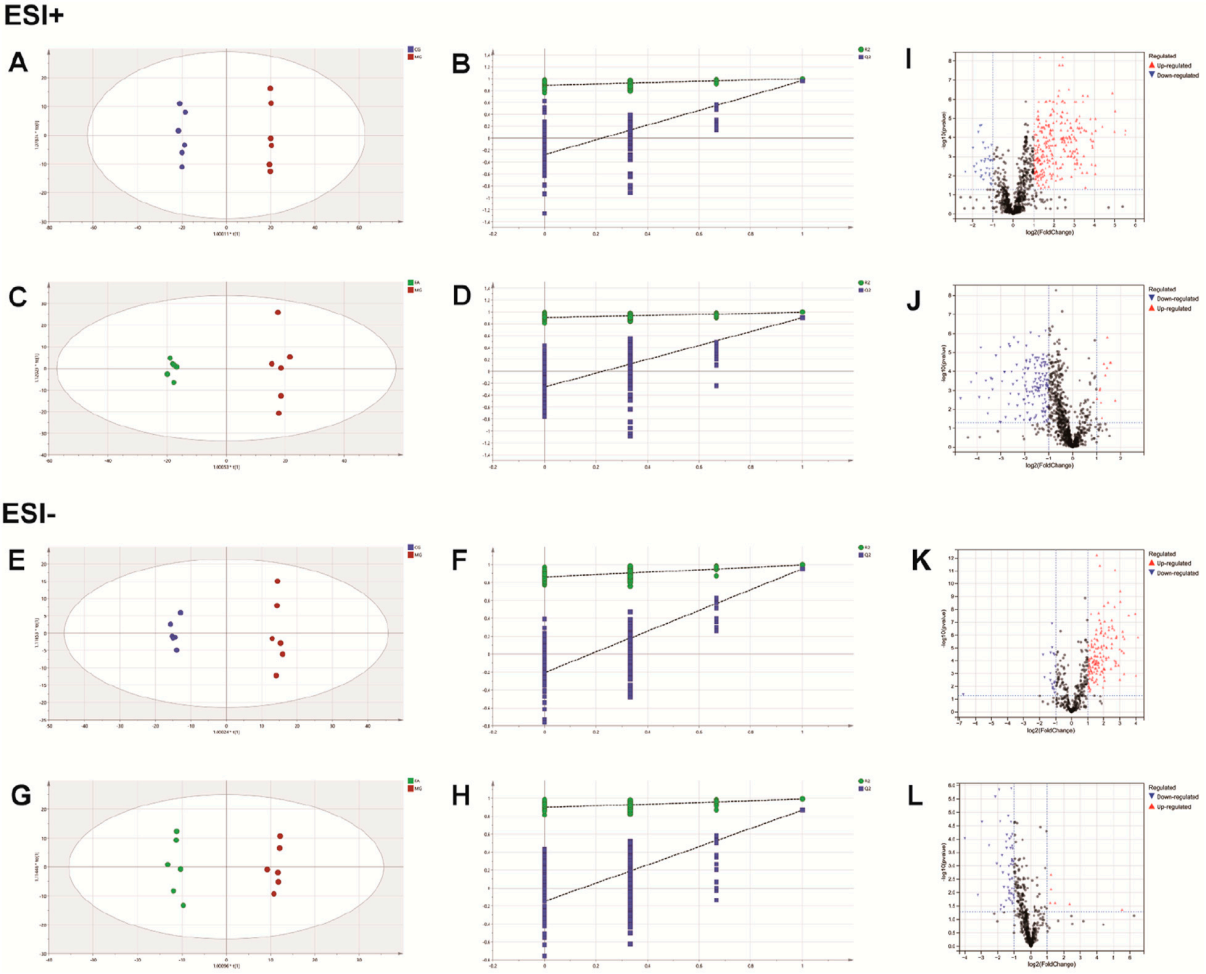
FA intervention on hyperlipidemia mice. Positive ESI model: Score plot of OPLS-DA between MG and CG **(A)**. The result of cross-validation of OPLS-DA model between MG and CG **(B)**. Score plot of OPLS-DA between FA and MG **(C)**. The result of cross-validation of OPLS-DA model between FA and MG **(D)**. Negative ESI model: Score plot of OPLS-DA between MG and CG **(E)**. The result of cross-validation of OPLS-DA model between MG and CG **(F)**. Score plot of OPLS-DA between FA and MG **(G)**. The result of cross-validation of OPLS-DA model between FA and MG **(H)**. Positive ESI model: The volcano plot of the differential metabolites was screened in the MG and CG **(I)**. The volcano plot of the differential metabolites was screened in the FA and MG **(J)**. Negative ESI model: The volcano plot of the differential metabolites was screened in the MG and CG **(K)**. The volcano plot of the differential metabolites was screened in the FA and MG **(L)**.

The PCA analysis (Figure 3) delineated distinct clusters for CG, MG, and FA in both positive and negative ion modes. Oral administration of FA effectively attenuated serum biochemical abnormalities induced by triton WR-1339 in hyperlipidemic mice (MG). Subsequently, OPLS-DA models were constructed for CG vs MG and MG vs FA comparisons, respectively. Figures 4A–H depict the OPLS-DA score plots along with results from the 200 permutation tests. Table 1 presents detailed model parameters including R^2^Y, Q^2^, and CV-ANOVA (*p*-values), which assess the model’s goodness of fit and predictability (Yan et al., 2022). Figure 4 showed that the serum metabolomics variables were evaluated with a suitable fit degree and high prediction ability. Additionally, differential metabolites were identified using volcano plots (Figures 4I–L). A total of 31 differential metabolites were discerned based on criteria set at VIP >1, *p*-value <0.05, FC > 1.2, or FC < 0.8 (Table 2). These findings highlight specific metabolic changes associated with FA treatment in hyperlipidemia, providing valuable insights into its therapeutic effects.

**TABLE 1:** The parameters of the OPLS-DA model.

Mode	Group	R^2^X	R^2^Y	Q^2^	CV-ANOVA
Positive	MG vs CG	0.513	0.998	0.965	3.61e^−5^
	FA vs MG	0.477	0.993	0.899	1.34e^−3^
Negative	MG vs CG	0.568	0.996	0.951	1.11e^−4^
	FA vs MG	0.420	0.991	0.866	3.53e^−3^

**TABLE 2:** Identification and trend of potential biomarkers.

No.	Metabolites	HMDB ID	Ion mode	Trend	
				MG vs CG	FA vs MG
1	L-Acetylcarnitine	HMDB0000201	ESI+	↓^##^	↑^**^
2	Palmitic acid	HMDB0000220	ESI+	↑^##^	↓^**^
3	α-Linolenic acid	HMDB0001388	ESI+	↑^##^	↓^**^
4	9-OxoODE	HMDB0004669	ESI+	↑^##^	↓^**^
5	5-HETE	HMDB0011134	ESI+	↑^##^	↓^**^
6	9(S)-HPETE	METPA1147	ESI+	↑^##^	↓^**^
7	Oleoyl glycine	HMDB0013631	ESI+	↑^##^	↓^**^
8	Palmitoleic acid	HMDB0003229	ESI+	↓^##^	↑^**^
9	γ-Tocotrienol	HMDB0012958	ESI+	↑^##^	↓^**^
10	N-Oleoyl phenylalanine	HMDB0062336	ESI+	↑^##^	↓^**^
11	2-Phytyl-1,4-naphthoquinone	HMDB0004649	ESI+	↑^##^	↓^**^
12	Ursolic acid	HMDB0002395	ESI+	↑^##^	↓^**^
13	LysoPA (P-16:0/0:0)	HMDB0011154	ESI+	↑^##^	↓^**^
14	N-Acetylglutamic acid	HMDB0001138	ESI+	↑^##^	↓^**^
15	LysoPC(18:0)	HMDB0011149	ESI+	↑^##^	↓^**^
16	Janthitrem C	HMDB0040684	ESI+	↑^##^	↓^**^
17	Janthitrem B	HMDB0030528	ESI+	↑^##^	↓^**^
18	9-Oxohexadecanoic acid	HMDB0030973	ESI-	↑^##^	↓^**^
19	3-Hydroxyhexadecanoic acid	HMDB0061658	ESI-	↑^##^	↓^**^
20	13-HODE	HMDB0004667	ESI-	↑^##^	↓^**^
21	8(R)-HPODE	HMDB0004706	ESI-	↑^##^	↓^**^
22	12,13-EpOME	HMDB0004702	ESI-	↑^##^	↓^**^
23	CE (16:0)	HMDB0000885	ESI-	↑^##^	↓^**^
24	(E)-Casimiroedine	HMDB0030274	ESI-	↑^##^	↓^**^
25	L-Methionine	HMDB0000696	ESI-	↑^##^	↓^**^
26	Arachidonic acid	HMDB0001043	ESI-	↑^##^	↓^**^
27	Leukotriene E3	HMDB0002355	ESI-	↑^##^	↓^**^
28	Dynorphin A (6-8)	HMDB0012932	ESI-	↑^##^	↓^**^
29	Linoleic acid	HMDB0000673	ESI-	↑^##^	↓^**^
30	Elaidic acid	HMDB0000573	ESI-	↑^##^	↓^**^
31	Hippuric acid	HMDB0000714	ESI-	↑^##^	↓^**^

### 3.3 Metabolic pathway of potential biomarkers

To investigate potential FA biomarkers for the therapy of hyperlipidemia and associated metabolic pathways, MetaboAnalyst 6.0 was utilized. Ten relevant metabolic pathways were identified using metabolic pathway enrichment analysis. The metabolic pathways with the strongest correlations (raw *p* < 0.05 and pathway impact > 0.05) (Xiao et al., 2023) were as follows: linoleic acid metabolism, arachidonic acid metabolism, and ether lipid metabolism. The metabolic pathway showed that FA exerts its lipid-lowering effect mainly regulates lipid and amino acid metabolism.

**FIGURE 5:**
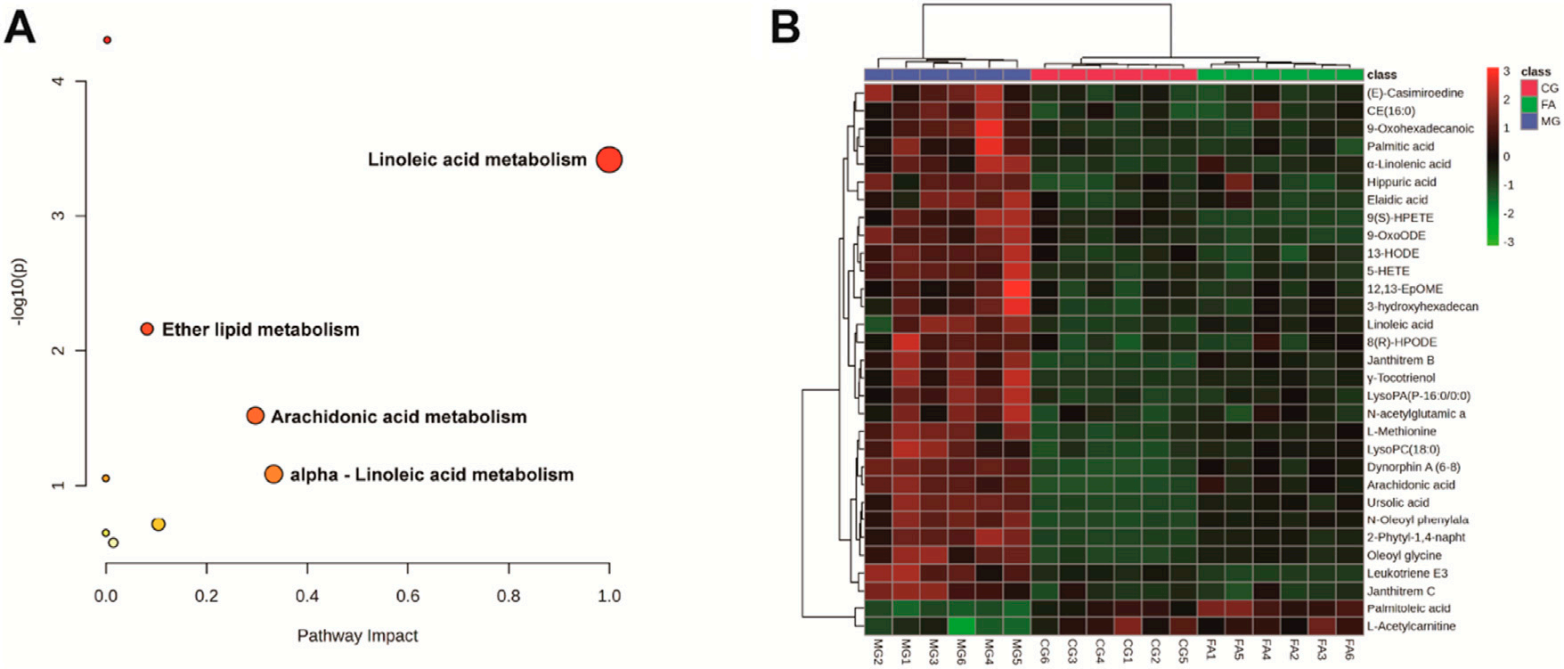
Metabolic pathway analysis according to the identified metabolites associated with hyperlipidemia **(A)**. Hierarchical clustering heatmap of the 31 differential metabolites with the degree of variation marked in red (upregulated) and green (downregulated) **(B)**.

**FIGURE 6:**
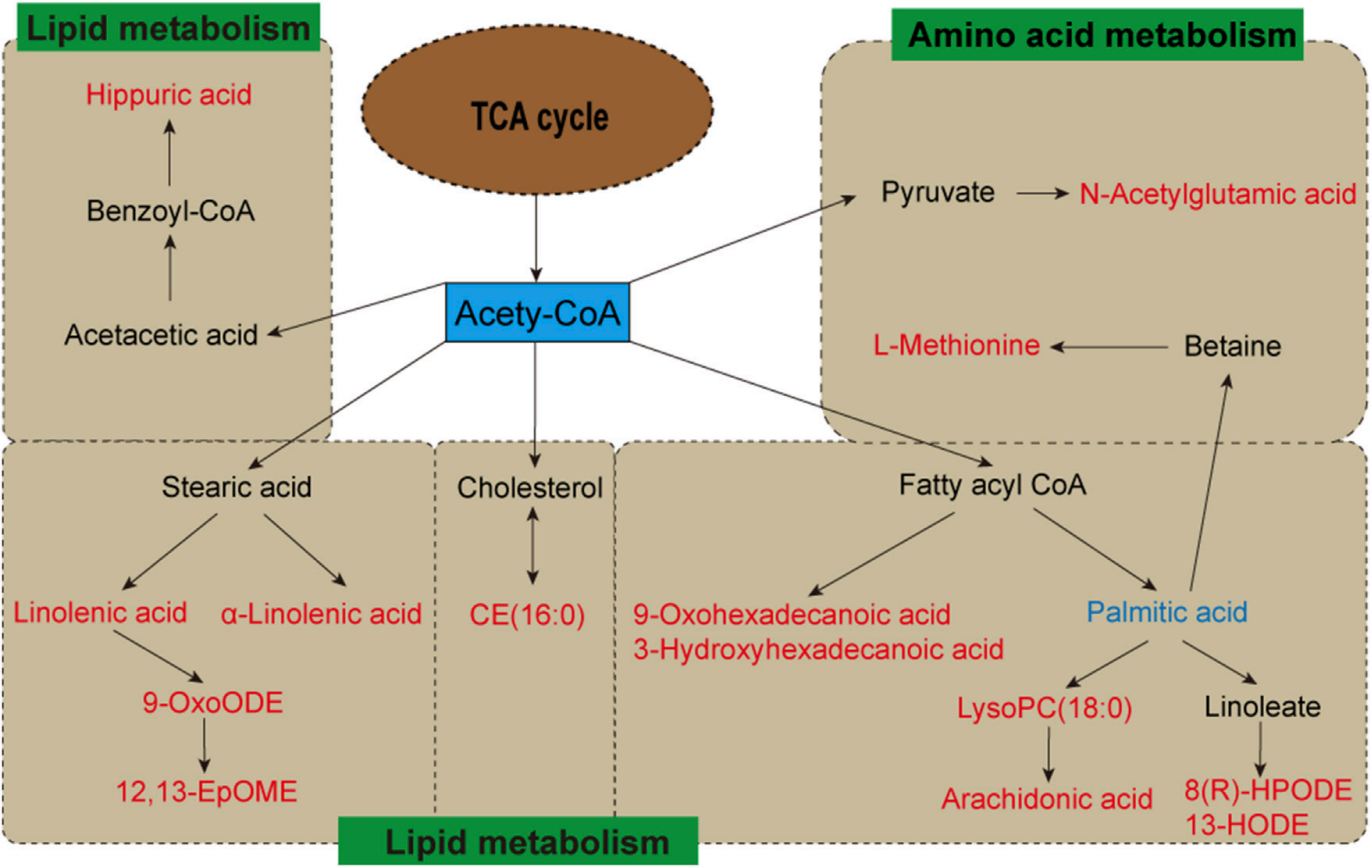
Metabolic pathways of potential biomarkers from metabolomics analysis. Metabolites with red markers show a significant increase in MG compared to CG but a decrease in FA compared with MG. Metabolites with blue markers indicate decreased levels of MG and an increase in FA.

The heatmap analysis visualized the differential metabolites in the three groups and revealed the correlation between them. Our results showed that 2 metabolites (L-acetylcarnitine and palmitoleic acid) were significantly downregulated in the MG compared to the CG. In addition, 29 metabolites were significantly upregulated in the MG compared to the CG, including 9-OxoODE, 12,13-EpOME, α-linolenic acid, hippuric acid, linoleic acid, etc. The levels of palmitic acid, α-linolenic acid, 5-HETE, N-oleoyl phenylalanine, LysoPC(18:0), N-acetylglutamic acid, janthitrem C, 12,13-EpOME, CE (16:0), L-methionine, arachidonic acid, and hippuric acid were significantly decreased in the CG, and the levels of these metabolites in FA were reversed and returned to normal or near-normal levels compared to MG. Therefore, they are considered potential biomarkers for the hypolipidemic effects of FA. Figure 5 and Table 2 showed the trend of biomarker levels in the three groups, which indicated a significant difference in the potential biomarkers between CG and MG, and FA could reverse these differences. Furthermore, metabolic pathways associated with these biomarkers were constructed based on the KEGG database (Figure 6). These analyses underscore FA’s ability to modulate specific metabolic pathways crucial for lipid regulation, offering mechanistic insights into its therapeutic potential for hyperlipidemia.

### 3.4 Network pharmacology

#### 3.4.1 Prediction of the targets

**FIGURE 7:**
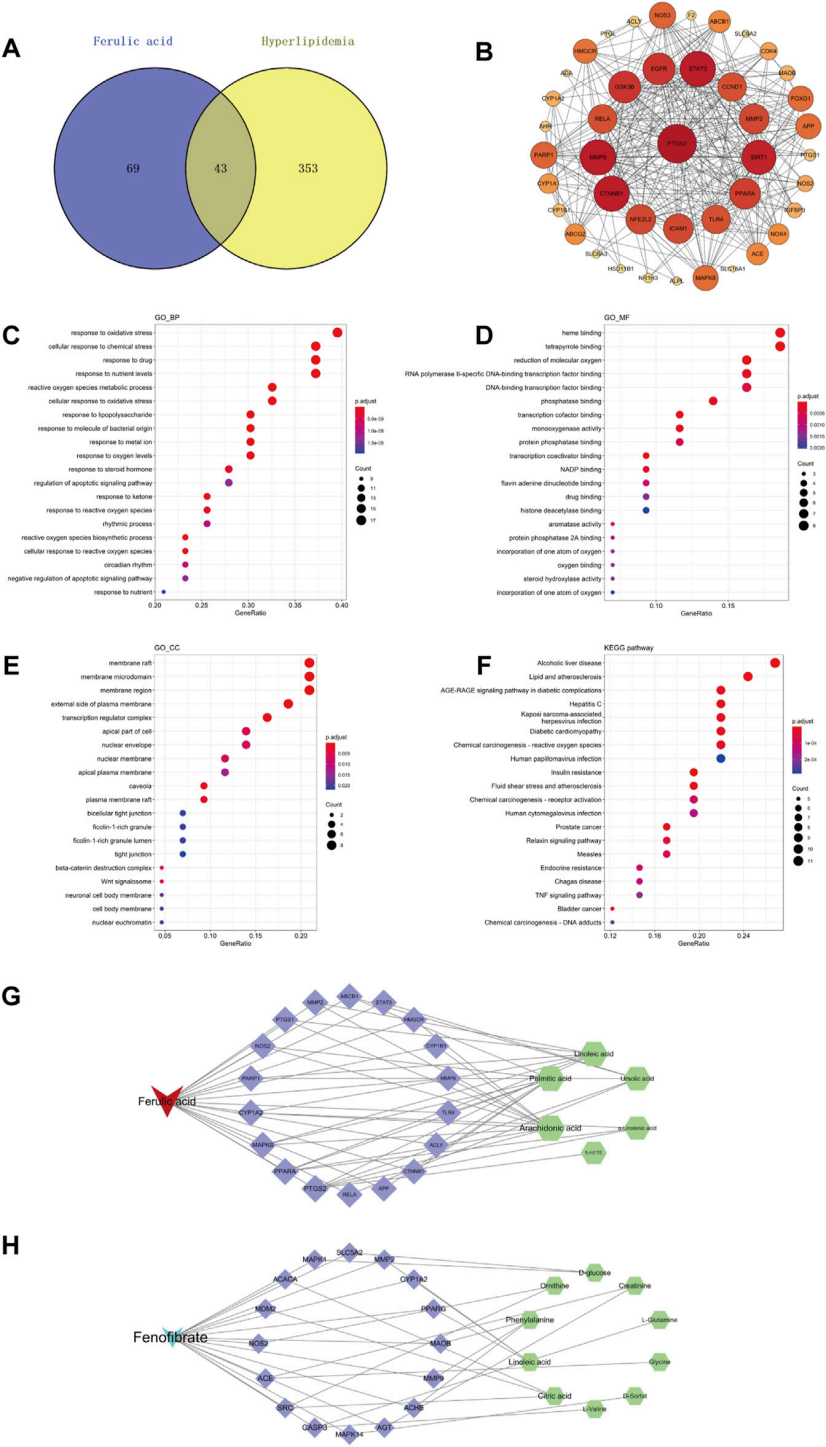
Network pharmacology of FA in the treatment of hyperlipidemia. Venny diagram of FA and hyperlipidemia intersection targets **(A)**. PPI network of potential targets **(B)**. GO and KEGG enrichment analysis of potential targets for FA to treat hyperlipidemia, biological processes **(C)**, molecular functions **(D)**, Cellular components **(E)**, and KEGG pathways **(F)**. Compound-target-metabolite (C-T-M) network of FA **(G)**, Compound-target-metabolite (C-T-M) network of fenofibrate **(H)**.

In this study, we searched the ChEMBL database for targets of FA using “ferulic acid” as the keyword and retrieved 112 targets. We also retrieved 258 targets from ChEMBL database using “fenofibrate” as the keyword. 396 targets with high relevance to hyperlipidemia were collected using GeneCard, CTD, and Drugbank databases. The overlapping targets between ferulic acid and hyperlipidemia in the Venny diagram (Figure 7A) revealed 43 potential targets between FA and hyperlipidemia. In addition, the overlapping targets in the Venny diagram (Supplementary Figure S2) revealed 40 potential targets between fenofibrate and hyperlipidemia.

#### 3.4.2 Construction PPI network

To further explore the relationship between protein and protein, we constructed a protein-protein interaction (PPI) network in the STRING database with the organism set as “*Homo sapiens*”, resulting in a total of 43 nodes, 306 edges, and an average node degree of 14.2 by setting the “confidence level” threshold higher than 0.4 (Figure 7B). The top 10 core targets of degree were based on the results of the degree value sorting, which were PTGS2, CTNNB1, MMP9, STAT3, SIRT1, GSK3B, EGFR, PPARA, NFE2L2, and ICAM1.

#### 3.4.3 GO and KEGG enrichment analysis

The software R was introduced to perform GO analysis of 43 potential targets, restricting the species to *Homo sapiens*. As a result, a total of 1,005 statistically significant GO entries were generated, including 906 biological processes (BP), 28 cellular components (CC), and 71 molecular functions (MF). The top 20 entries with the lowest *p* values in BP, CC, and MF are presented in Figures 7C–E. In addition, we conducted KEGG enrichment analysis on 43 potential targets using R to identify pathways highly relevant to FA and hyperlipidemia. After analyzing 101 pathways in KEGG, the top 20 most relevant signaling pathways were presented in Figure 7F.

Notably, many genes identified through GO analysis involved key regulatory processes, such as oxidative stress, oxygen metabolic/biosynthetic processes, and apoptotic signals regulation. These potential genes were also associated with the binding of oxidation-related substances, transcription factor binding, and regulating the activity of related enzymes (e.g., monooxygenase, aromatase, steroid hydroxylase, etc.). The KEGG enrichment analysis identified potential targets in several pathways, including alcoholic liver disease, lipid and atherosclerosis-related pathways, insulin resistance, TNF signaling pathway, etc.

### 3.5 Conjoint analysis

To further explore the hypolipidemic mechanism of FA on hyperlipidemia, we integrated metabolomics and network pharmacology to find core targets for treating hyperlipidemia with FA. We constructed a C-T-M network using Cytoscape 3.7.2, the results were shown in Figure 7G. A total of 18 were considered as core targets, including PTGS2, PPARA, MAPK8, CYP1A2, PARP1, NOS2, PTGS1, MMP2, ABCB1, STAT3, HMGCR, CYP1B1, MMP9, TLR4, ACLY, CTNNB1, APP, and RELA. Furthermore, arachidonic acid, palmitic acid, linoleic acid, ursolic acid, α-linolenic acid, and 5-HETE were recognized as potential biomarkers for FA treatment of hyperlipidemia. These findings were supported by KEGG pathway enrichment analysis, which highlighted FA’s role in modulating lipid and atherosclerosis-related pathways, consistent with our metabolomics results. This integrated approach underscores FA’s multifaceted therapeutic effects in addressing hyperlipidemia by regulating key metabolic pathways and targets, providing a comprehensive understanding of its therapeutic potential.

Moreover, we also constructed a C-T-M network of fenofibrate, the results were shown in Figure 7H. A total of 16 were considered as core targets, including ACACA, MDM2, SRC, NOS2, CASP3, MAPK14, ACE, AGT, ACHE, MMP9, MAOB, PPARG, CYP1A2, MMP2, SLC5A2, and MAPK1. Furthermore, citric acid, ornithine, L-glutamine, L-valine, D-sorbit, glycine, creatinine, phenylalanine, linoleic acid, and D-glucose were recognized as potential biomarkers for fenofibrate treatment of hyperlipidemia.

A comparative analysis of the combined effects of FA and fenofibrate revealed four common targets: CYP1A2, MMP2, MMP9, and NOS2. Furthermore, FA was found to influence the expression of the PPARA gene, while fenofibrate modulated the expression of the PPARG gene. These findings elucidate that FA targets PPARα, whereas fenofibrate targets PPARγ, to exert their lipid-lowering effects.

### 3.6 Molecular docking

We explored the interaction between FA and core targets (PTGS2, PPARA, MAPK8, CYP1A2, PARP1, NOS2, PTGS1, MMP2, ABCB1, STAT3, HMGCR, CYP1B1, MMP9, TLR4, ACLY, CTNNB1, APP, and RELA) using molecular docking analysis. Using AutoDock Vina software, 18 docking results were obtained and showed low binding energies, indicating a strong affinity between the FA and core targets. It was worth noting that all core targets demonstrated high affinity binding to FA. The results were shown in Table 3, and all of them were less than −3 kcal/mol, and the lower binding energy indicates a more stable binding of FA to the target. The results suggested that FA can spontaneously bind to each target via hydrogen bonding, van der Waals forces, etc., indicating their important roles in the molecular mechanism of FA with hyperlipidemia.

**TABLE 3:** Results of 18 core targets and FA of molecular docking.

No.	Molecule	Target	PDB ID	Binding affinity (kcal/mol)
1	Ferulic acid	PTGS2	5F19	−3.5
2	Ferulic acid	PPARA	3ET1	−6.5
3	Ferulic acid	MAPK8	4G1W	−5.9
4	Ferulic acid	CYP1A2	2HI4	−6.2
5	Ferulic acid	PARP1	7ONS	−6.1
6	Ferulic acid	NOS2	3EJ8	−7.2
7	Ferulic acid	PTGS1	6Y3C	−4.9
8	Ferulic acid	MMP2	8H78	−5.1
9	Ferulic acid	ABCB1	6FN1	−4.1
10	Ferulic acid	STAT3	6NJS	−5.0
11	Ferulic acid	HMGCR	3BGL	−5.9
12	Ferulic acid	CYP1B1	6IQ5	−6.8
13	Ferulic acid	MMP9	1ITV	−5.5
14	Ferulic acid	TLR4	4G8A	−4.8
15	Ferulic acid	ACLY	6UI9	−4.9
16	Ferulic acid	CTNNB1	1QZ7	−5.7
17	Ferulic acid	APP	3L3T	−3.1
18	Ferulic acid	RELA	2VGE	−6.3

## 4 Discussion

In this study, a triton WR-1339-induced hyperlipidemic mouse model was used to evaluate the lipid-lowering effect of FA. We determined the biochemical indices in serum and clarified that FA improved lipid levels and reduced hepatic lipid accumulation and inflammatory infiltration in hyperlipidemic mice. Subsequently, metabolomics was used to explore potential biomarkers for FA regulation of hyperlipidemia, 31 metabolites were identified as potential biomarkers. Ultimately, combining metabolomics and network pharmacology, 18 core targets were identified as therapeutic targets for FA in treating hyperlipidemia.

In clinical practice, hyperlipidemia is diagnosed by assessing lipid indices (TC, TG, HDL-c, and LDL-c) lipid indices (Ta et al., 2023). Our study showed that TC, TG, and LDL-c levels were significantly increased, and HDL-c was decreased in triton WR-1339-induced hyperlipidemia mice. In contrast, after treatment with FA or fenofibrate, TC, TG, and LDL-c declined, and HDL-c was increased, demonstrating that both FA and fenofibrate exert lipid-lowering effects. The results of H&E were consistent with the results of biochemical indices, indicating that FA has a hypolipidemic effect.

The total cholesterol levels in mice with hyperlipidemia were elevated through intravenous or subcutaneous injection of Triton WR 1339. This induction might occur due to its impact on cholesterol metabolism, potentially leading to hyperlipidemia by enhancing lipid synthesis, hindering their degradation or excretion, or a combination of these mechanisms (Hirsch and Kellner, 1956). The research indicated that blood cholesterol levels in rats tripled 24 h following the administration of Triton WR 1339 but subsequently declined after 72 h. Concurrently, cholesterol concentrations in the liver increased, while blood cholesterol levels returned to baseline (Frantz and Hinkelman, 1955). Previous studies (Li et al., 2020; Xiao et al., 2023) have shown that Triton WR-1339 can prompt rapid lipid accumulation in the livers of mice, which aligns with our observations. Furthermore, Triton WR-1339 has been reported to elevate oxidative stress in mice. Significant increases in lipid peroxidation and protein carbonylation were observed in the livers of Triton WR-1339-induced hyperlipidemic mice (Ramos et al., 2021). Similar oxidative stress elevation has been documented in hyperlipidemic rats treated with Triton WR-1339 (Khlifi et al., 2019).

Metabolomics is an approach that analyzes metabolites in organisms with a relative molecular mass of less than 1,000 to explain the relative relationship between the metabolites and physiological-pathological changes and is an integral part of systems biology (Lei et al., 2011). Evidence shows that metabolic pathways in hyperlipidemic mice involve three pathways: lipid metabolism, energy metabolism, and amino acid metabolism (Yan et al., 2022). According to our metabolomics analysis, the main causes of the development of hyperlipidemia are abnormalities in lipid metabolism and amino acid metabolism, this is consistent with the pathways enriched in HFD-induced hyperlipidemic mice (Du et al., 2022), suggesting that the triton WR-1339-induced hyperlipidemic mouse model is credible. We determined the levels of some metabolites associated with lipid metabolism and amino acid metabolism in the serum of mice via metabolomics. The enrichment analysis showed the main metabolic pathways, including linoleic acid metabolism, arachidonic acid metabolism, and ether lipid metabolism.

Linoleic acid has been shown to ameliorate high-fat-induced insulin resistance and the ratio of a-linoleic acid to linoleic acid modulates lipid metabolism levels in obese mice (Wang and Wang, 2023; Wu et al., 2024). Interestingly, replacing linoleic acid with α-linoleic acid in the diet of people at high risk for coronary heart disease decreased diastolic blood pressure and increased serum triacylglycerol concentrations (Bemelmans et al., 2000). Evidence shows that arachidonic acid is associated with dyslipidemia and cholesterol-associated lipoprotein metabolism and that arachidonic acid increased plasma concentrations of TC and ApoB, as well as elevated ApoB protein expression and decreased protein expression of ABCG5/8 in mice (Li et al., 2022). LDL and HDL inhibit platelet aggregation and arachidonic acid production in hypercholesterolemic rabbits (Aslam et al., 2008). Arachidonic acid metabolism is involved in cardiac regulation in prediabetic rats (Miklankova et al., 2021). Surprisingly, the arachidonic acid metabolic pathway inhibits renal cell carcinoma proliferation (Liu et al., 2022). One study found differences in ether lipid metabolism between the ginsenoside Rb1 administration group and hyperlipidemic mice, suggesting that ginsenoside Rb1 can inhibit hyperlipidemia by regulating ether lipid metabolism (Jia et al., 2021). In addition, ether lipid metabolic activity is strongly associated with metabolic syndrome and hyperlipidemia (Gao et al., 2019).

Furthermore, the mechanisms associated with FA against hyperlipidemia were explored by network pharmacology, and KEGG results showed that FA treatment of hyperlipidemia involves a lipid and atherosclerosis-related pathway. Combining network pharmacology and metabolomics, six metabolites (arachidonic acid, palmitic acid, linoleic acid, ursolic acid, α-linolenic acid, and 5-HETE) were identified as biomarkers for FA treating hyperlipidemia, and 18 targets were identified as core targets for FA against hyperlipidemia.

The analysis of the C-T-M network in Figures 7G, H indicates that FA and fenofibrate, both used in the treatment of hyperlipidemia, have several overlapping targets, including CYP1A2, MMP2, MMP9, and NOS2. Interestingly, PPARA is implicated in the effects of FA, whereas PPARG is involved in those of fenofibrate. Additionally, the interaction with inflammatory targets suggests that the lipid-lowering effect of FA may also involve anti-inflammatory pathways (Rehman et al., 2019).

Finally, we used molecular docking for further validation to confirm whether FA could act on the 18 core targets. FA inhibits PTGS2 protein expression and exerts anti-angiogenic effects (Ekowati et al., 2020; Kumar and Pruthi, 2015), it also has good infinity with AMPK8 and CTNNB1 targets (Zhou et al., 2022). CYP1A2 and CYP3A4 are human cytochrome P450 enzyme isoforms that mediate FA metabolism (Zhuang et al., 2017). FA improves rat spermatogenesis by reducing radiation-induced testicular damage by inhibiting PARP1 protein expression (El-Mesallamy et al., 2018) and inhibits LPS-induced neuroinflammatory mitochondrial apoptotic signaling molecules (caspase-3 and PARP1) in the mouse brain, also interferes with the TLR4/MD2 complex-binding site to block the process of neuroinflammation induced through microglia activation (Rehman et al., 2019). FA-induced decreased expression levels of BACE1 and APP and increased expression of MMP2 and MMP9 were found by cellular experiments, and FA may be a potential herbal component for the treatment of Alzheimer’s disease (Meng et al., 2018). In addition, FA significantly downregulated ABCB1 expression in a concentration-dependent manner, as its chemosensitizing effect reduced paclitaxel resistance in cells (Muthusamy et al., 2016). Nevertheless, further experimental validation is needed to determine whether FA acts on the core targets.

## 5 Conclusion

In conclusion, combining network pharmacology and metabolomics was used to explore the lipid-lowering mechanism of FA in hyperlipidemic mice induced by a triton WR-1339. In addition, 31 differential metabolites related to FA against hyperlipidemia were screened, including lipid and amino acid metabolism. After administrating with FA, its main involvement in improving metabolic disorders and maintaining the dynamic balance of metabolites is associated with lipid metabolism, including linoleic acid metabolism, arachidonic acid metabolism, and ether lipid metabolism. Finally, we identified 18 core targets and 6 biomarkers by combined network pharmacology and metabolomics analysis. Our study investigated the mechanism of FA in treating hyperlipidemia, elucidated the relationship between metabolites and lipid-lowering, and provided a scientific basis for clinical application and promotion.

## Data Availability

All untargeted metabolomic data used in this publication have been deposited to the EMBL-EBI MetaboLights database with the identifier MTBLS11067. The complete data set can be accessed at https://www.ebi.ac.uk/metabolights/MTBLS11067.
