# Supplementary material for: Metabolomics and network pharmacology reveal partial insights into the hypolipidemic mechanisms of ferulic acid in a dyslipidemia mouse model
Source: Front Pharmacol. 2024 Sep 20;15:1466114. doi: 10.3389/fphar.2024.1466114 (PMC11453126; doi:10.3389/fphar.2024.1466114)
Supplement: Supplementary file 1 [file DataSheet1.doc]

**Supplementary information**

1. The serum metabolomics analysis was carried out by Agilent 1290 II coupled to AB SCIEX X500R Q-TOF-MS/MS. The chromatographic separations were achieved on a Waters UPLC BEH C18 column (2.1 mm × 100 mm, 1.7 μm), flow rate 0.3 mL/min, injector temperature 4 °C, column temperature 35°C, injection volume 1 μL. Mobile phase: gradient elution of acetonitrile (A) - 0.1% formic acid water (B) (0-1 min, 2% A; 1-3 min, 2%-10% A; 3-7 min, 10%-40% A; 7-16 min, 40%-75% A; 16-20 min, 75%-98% A; 20-23 min, 98% A). MS was performed in positive and negative ion modes with electrospray ionization (ESI). The optimization source parameters were set as follows: ion voltage -4,500 V and +5,500 V, Gas1 55 psi, Gas2 55 psi, curtain gas 35 psi, de-clustering potential voltage 60 V, ion source temperature 500 °C, collision energy 35 V, collision energy spread 15 V, full scan *m*/*z* 50-1,000. The TIC chromatograms are shown in Figure S1.





FIGURE S1. Determination of serum metabolites by UPLC-QTOF-MS/MS. (A) TIC chromatograms of serum metabolites in positive (A) and negative (B) ion modes.


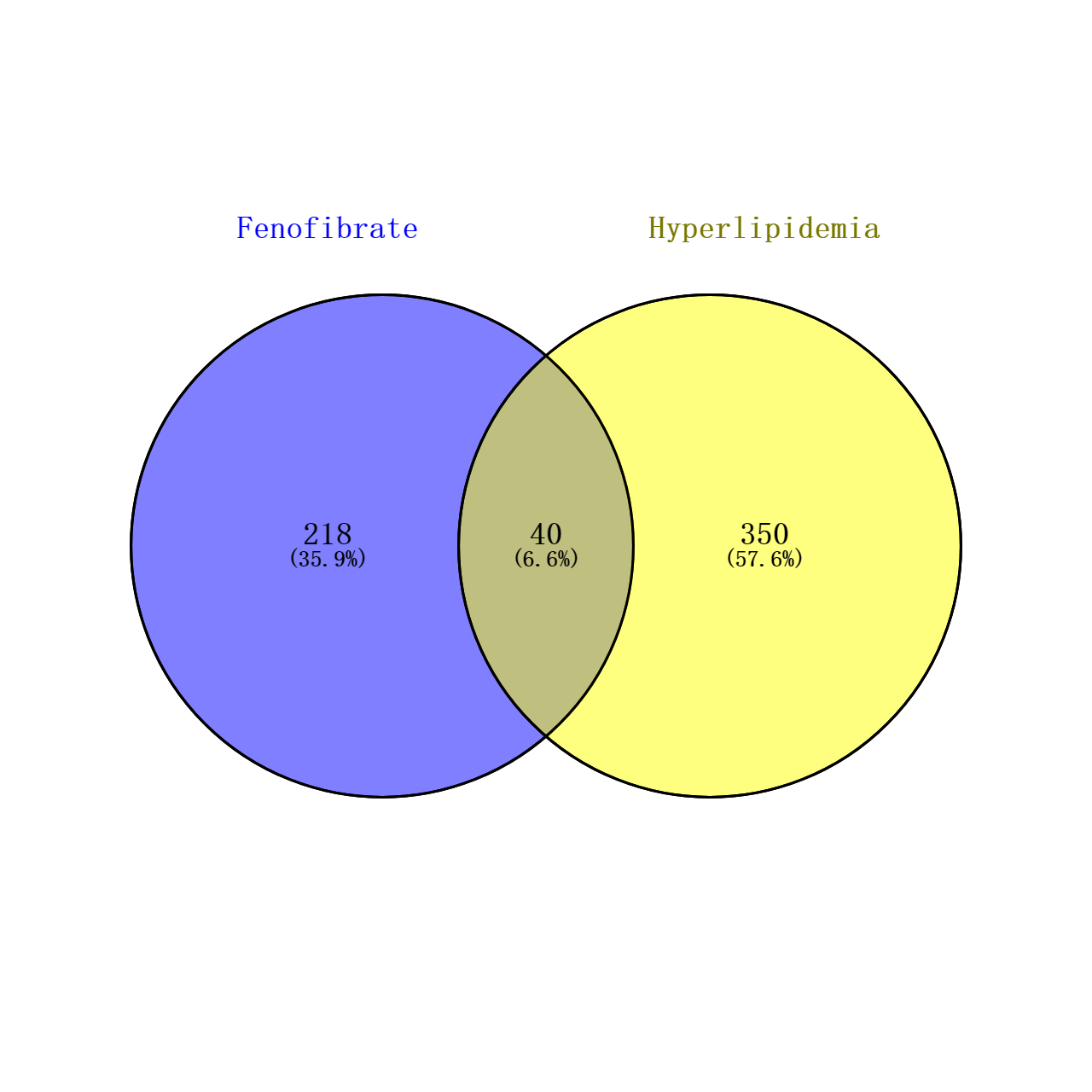
2. Venny 2.1.0 (https://bioinfogp.cnb.csic.es/tools/venny/) screened the overlapping targets between fenofibrate and hyperlipidemia. We obtained 40 overlapped targets, the results were showen in Figure S2.

FIGURE S2. Venny diagram of fenofibrate and hyperlipidemia intersection targets.

3. We collected the metabolomic data between fenofibrate group and normal control group from some literatures (Lu et al., 2011; Xu et al., 2014), a total of 35 metabolites were obtained, the metabolites were shown in Table S1.

TABLE S1 The metabolites between fenofibrate group and normal control group

| No. | Metabolites | Reference |
| --- | --- | --- |
| 1 | β-hydroxybutyrate | Lu et al., 2011 |
| 2 | Ribose | Lu et al., 2011 |
| 3 | C18:0 | Lu et al., 2011 |
| 4 | C20:0 | Lu et al., 2011 |
| 5 | C18:2n-6/7 | Lu et al., 2011 |
| 6 | C18:3n-3 | Lu et al., 2011 |
| 7 | C18:3n-6 | Lu et al., 2011 |
| 8 | C20:2n-6 | Lu et al., 2011 |
| 9 | C20:3n-6 | Lu et al., 2011 |
| 10 | C20:3n-9 | Lu et al., 2011 |
| 11 | C20:4n-3 | Lu et al., 2011 |
| 12 | C20:4n-6 | Lu et al., 2011 |
| 13 | C22:4n-6 | Lu et al., 2011 |
| 14 | C22:5n-3 | Lu et al., 2011 |
| 15 | C22:5n-6 | Lu et al., 2011 |
| 16 | C22:6n-3 | Lu et al., 2011 |
| 17 | Galactose | Xu et al., 2014 |
| 18 | Mannose | Xu et al., 2014 |
| 19 | D-sorbit | Xu et al., 2014 |
| 20 | D-glucose | Xu et al., 2014 |
| 21 | Citric acid | Xu et al., 2014 |
| 22 | L-glutamine | Xu et al., 2014 |
| 23 | Creatinine | Xu et al., 2014 |
| 24 | β-hydroxybutyric acid | Xu et al., 2014 |
| 25 | Myo-inositol | Xu et al., 2014 |
| 26 | Glycine | Xu et al., 2014 |
| 27 | Tyrosine | Xu et al., 2014 |
| 28 | Ornithine | Xu et al., 2014 |
| 29 | Phenylalanine | Xu et al., 2014 |
| 30 | Proline | Xu et al., 2014 |
| 31 | Isoleucine | Xu et al., 2014 |
| 32 | L-valine | Xu et al., 2014 |
| 33 | Alanine | Xu et al., 2014 |
| 34 | Cholesterol | Xu et al., 2014 |
| 35 | Linoleic acid | Xu et al., 2014 |

**Reference**

Lu Y, V. BM, S. W, M. M and Kersten S (2011) Comparative transcriptomic and metabolomic analysis of fenofibrate and fish oil treatments in mice. *Physiol Genomics* **43**:1307-1318.

Xu QY, Liu YH, Zhang Q, Ma B, Yang ZD, Liu L, Yao D, Cui GB, Sun JJ and Wu ZM (2014) Metabolomic analysis of simvastatin and fenofibrate intervention in high-lipid diet-induced hyperlipidemia rats. *Acta Pharmacol Sin* **35**:1265-1273.
